# Data in support of large scale comparative codon usage analysis in *Leishmania* and Trypanosomatids

**DOI:** 10.1016/j.dib.2015.06.003

**Published:** 2015-06-18

**Authors:** Abhishek Subramanian, Ram Rup Sarkar

**Affiliations:** a Chemical Engineering and Process Development, CSIR-National Chemical Laboratory, Pune, Maharashtra, India; b Academy of Scientific & Innovative Research (AcSIR), CSIR-NCL Campus, Pune, India

## Abstract

This data article contains data related to the article “*Comparison of codon usage bias across Leishmania and Trypanosomatids to understand mRNA secondary structure, relative protein abundance and pathway functions*” by Subramanian and Sarkar, Genomics, 2015 (10.1016/j.ygeno.2015.05.009). The data comprises of sequence-based measures that quantify the effect of codon usage across genomes. The data thus generated represents computed values of codon usage indices like relative synonymous codon usage (RSCU), effective number of codons (ENC), and codon adaptation index (CAI), a set of single copy orthologous genes common to the 13 Trypanosomatids, and comparisons of CAI between genes of different functions. This forms a basis of comparison to infer the causes and consequences of codon usage bias in *Leishmania* and other Trypanosomatids.

## 1 Specifications table

**Table:** 

Subject area	*Biology*
More specific subject area	*Comparative and functional Genomics*
Type of data	*Table*
How data was acquired	*Data for codon usage comparison was computationally generated from raw coding sequence data using established software programs and in-house PERL codes designed for specific purposes*
Data format	*Analyzed*
Experimental factors	*N/A*
Experimental features	*All the coding sequences of 13 Trypanosomatid genomes (filtered to remove sampling error) have been used to compute codon usage indices like RSCU, degree of mutational pressure, ENC, CAI, codon and amino acid contexts*
Data source location	*Raw coding sequence data was collated from TriTrypDB database* v.8.1 [5].
Data accessibility	*Data available with this article*

## 2 Value of the data

• The data can be used to predict the relation of codon usage with GC content and protein expression, and its comparison between 13 Trypanosomatid species.
• The data presented here is useful to analyze the otherwise raw sequences and contains some useful information related to usage of codons within the considered genomes.
• The corresponding comparison of codon usage at the pathway level also helps to understand phenotypic variation in Leishmaniasis.
• The data helps in knowledge generation regarding putative differences that lead to species-specific clinical manifestations between *Leishmania* species.

## 3 Data, experimental design, materials and methods

The data presented here represent the computed values of codon usage indices like relative synonymous codon usage (RSCU) [2], effective number of codons (ENC) [3], and codon adaptation index (CAI) [2], a set of single copy orthologous genes common to the 13 Trypanosomatids, and comparisons of CAI between genes of different functions. CAI and ENC for each gene in every genome were computed using the EMBOSS package [4]. Single copy orthologous gene groups and predicted gene ontology (GO) processes for every gene were extracted from TriTrypDB database v.8.1 [5]. Single copy orthologous genes across the *Leishmania* species for comparison of CAI values were identified using the Proteinortho orthology detection tool v.5.10 [6].

## 4 Comparison of RSCUs between *Leishmania* and other Trypanosomatids

Relative Synonymous Codon Usage (RSCU) is a measure that represents the relative frequency of a codon within a genome. RSCU was computed for each codon within every considered genome. The first dataset (Supplementary Table S1) represents the RSCU values of each codon within the 13 Trypanosomatid genomes generated using in-house PERL codes. RSCU>1 for a particular codon indicates that the codon is much more frequent within a genome than expected and RSCU<1 denotes that the codon is less frequent within a genome. In Supplementary Table S1, codons with RSCU<1 are colored in blue whereas codons with RSCU>1 are colored in red. It can be clearly observed that the frequencies of CUG (Leu) and CGC (Arg) are considerably higher in *Leishmania* and *Crithidia* as compared to *Trypanosoma.* Also, specific codons (such as GUG – Val and CUG – Val) are preferred and certain codons (such UUA – Leu and GUA – Val) are avoided across all the 13 Trypanosomatid genomes [1].

## 5 Identification of single copy orthologous genes common for the 13 Trypanosomatids

To calculate the number of pairwise substitutions between genes of Trypanosomatid genomes, it was necessary to shortlist a set of 1218 single copy orthologous genes common to all the 13 Trypanosomatid species. For this purpose, we downloaded all possible orthologous groups between the 13 Trypanosomatids from TriTrypDB database v.8.1 [5] and manually refined the dataset for extraction of single copy orthologues. The second dataset (Supplementary Table S2) represents a manually curated dataset of single copy orthologous genes common to all the 13 Trypanosomatids.

## 6 ENC and CAI comparison between *Leishmania* and other Trypanosomatids

Effective Number of Codons (ENC) is a non-directional measure of codon usage bias (CUB) that is known to be dependent upon nucleotide composition of a gene. Codon Adaptation Index (CAI) is a directional measure of CUB, which quantifies the degree of translation selection acting upon a gene. Comparison of ENC and CAI across species can suggest more about CUB due to biased nucleotide compositions and translation selection [1]. ENC values are scaled between 20 and 61. A gene with ENC value of 20 indicates the effective biased usage of only one particular codon to code for an amino acid. ENC value close to 61 indicates the equal usage of all possible codons to code for a particular amino acid, suggesting no codon bias. The values of CAI are scaled between 0 and 1. CAI of a gene close to 1 suggests that the gene experiences a higher selection pressure to maintain a specific codon usage that is optimized for efficient translation. The third dataset (Supplementary Table S3) represents the ENC and CAI values of each gene within each Trypanosomatid genome.

## 7 Codon and amino acid contexts across Trypanosomatids

Efficiency of translation can be maintained through the presence of defined paired codon and amino acid contexts [7]. The fourth and fifth dataset (Supplementary Table S4 and S5) represent the frequencies of the codon and amino acid pairs within the 13 Trypanosomatids genomes generated from the coding sequences using in-house PERL codes. Codon context patterns reveal a high variability among Trypanosomatid species. Homogenous codon contexts having high GC content are mostly preferred in *Leishmania* whereas homogenous contexts having bias for AT content are preferred in *Trypanosoma* [1].

## 8 Codon usage bias and pathway level functions

CAI was demonstrated to be positively correlated to relative protein abundance in *Leishmania* [1]. Hence, assuming CAI to be a predictor of protein expression, we compared CAI values of genes belonging to different pathways/processes. For this comparison, we extracted a set of single copy orthologues common to all the 6 species of *Leishmania* considered in the study. The sixth dataset (Supplementary Table S6) represents percentage of genes belonging to a certain GO process having a high CAI (CAI>0.5) in *Leishmania* genomes. From this comparison, it could be observed that around 90% of genes in *Leishmania donovani* demonstrate highest CAI values when compared to other *Leishmania* species [1]. The seventh dataset (Supplementary Table S7) represents the variation in CAI values for the set of single copy orthologous genes common to the 6 *Leishmania* species. Enzymes of specific functions exhibit low to high variances across the *Leishmania* species [1].

## Footnotes

Appendix A Supplementary data associated with this article can be found in the online version at doi:10.1016/j.dib.2015.06.003.
